# Road traffic fatalities in selected governorates of Iraq from 2010 to 2013: prospective surveillance

**DOI:** 10.1186/s13031-016-0070-0

**Published:** 2016-02-24

**Authors:** Eva Leidman, Maret Maliniak, Abdul-Salam Saleh Sultan, Ahmed Hassan, Syed Jaffar Hussain, Oleg O. Bilukha

**Affiliations:** Emergency Response and Recovery Branch, Division of Global Health Protection, Center for Global Health, Centers for Disease Control and Prevention, 4770 Buford Hwy, MS F-60, Atlanta, GA 30341 USA; Department of Epidemiology, Rollins School of Public Health, Emory University, 1518 Clifton Road Northeast, Atlanta, GA 30322 USA; Human Resources Training and Development Center, Ministry of Health, Bab Al Mu’adham Street, Baghdad, Iraq; Medical Operations and Specialized Services Directorate, Operations Department, Ministry of Health, Al Adham Street, Baghdad, Iraq; World Health Organization, International Zone, UNAMI compound, Baghdad, Iraq

**Keywords:** Iraq, Injury, Conflict, Fatal, Road traffic, Surveillance

## Abstract

**Background:**

The insurgency tactics that characterize modern warfare, such as suicide car bombs and roadside bombs, have the potential to significantly impact road traffic injuries in conflict affected-countries. As road traffic incidents are one of the top ten causes of death in Iraq, changes in incidence have important implications for the health system. We aimed to describe patterns of road traffic fatalities for all demographic groups and types of road users in Iraq during a period characterized by a resurgence in insurgency activity.

**Methods:**

Iraqi Ministry of Health routine prospective injury surveillance collects information on all fatal injuries in eight governorates of Iraq: Baghdad, Al-Anbar, Basrah, Erbil, Kerbala, Maysan, Ninevah, and Al-Sulaimaniya. From all injury fatalities documented at the coroner office, we analyzed only those attributed to road traffic that occurred between 1 January 2010 and 31 December 2013. Coroners ascertain information from physical examinations, police reports and family members.

**Results:**

Analysis included 7,976 road traffic fatalities. Overall, 6,238 (78.2 %) fatalities were male and 2,272 (28.5 %) were children under 18 years of age. The highest numbers of road traffic fatalities were among males 15 to 34 years of age and children of both sexes under 5 years of age. 49.2 % of fatalities occurred among pedestrians. Among children and females, the majority of road traffic fatalities were pedestrians, 69.0 % and 56.6 %, respectively. Fatalities among motorcyclists (3.7 %) and bicyclists (0.4 %) were least common. Rates of road traffic fatalities ranged from 8.6 to 10.7 per 100,000 population.

**Conclusions:**

The injury surveillance system provides the first data from a conflict-affected country on road traffic fatalities disaggregated by type of road user. The highest numbers of fatalities were among children and young men. Nearly half of fatalities were pedestrians, a proportion nearly double that of any neighboring country. As insurgency activity increased in 2013, the number of road traffic fatalities declined.

## Background

An estimated 1.2 million people worldwide die from road traffic injuries (RTI) each year [1]. Road traffic is the eighth leading cause of death globally, ahead of diabetes, tuberculosis, malaria and all other injury categories [2]. Even in conflict-affected countries such as Afghanistan, Libya, Pakistan, and Yemen, road traffic remains the most common mechanism of injury fatality, causing between two and eight times more fatalities than forces of war and legal intervention [3]. The World Health Organization (WHO) estimates the traffic fatality rate in the Eastern Mediterranean Region (EMR) to be the second highest rate globally after the African Region, and increasing in several of the countries in the region [1, 4]. Iraq has the second highest road traffic fatality rate within the EMR [1].

Increasing rates of traffic fatalities in the EMR have been attributed to a lack of comprehensive road safety legislation, irregular road safety inspections, and rapid motorization [5]. Between 2007 and 2010, the number of registered vehicles in the EMR increased by 15 % to over 60 million vehicles [5, 6]. In Iraq, importation of vehicles spiked following the end of economic sanctions in 2003 and has continued to increase despite the fragile security situation [1, 7, 8]. Approximately one in ten people own a car in Iraq [9]. Despite the conflict, roads are more highly utilized today than at the beginning of the conflict. In 2003, an average of 200 to 500 vehicles per day were travelling on some of the major roads in Iraqi Kurdistan [10]. At present, the Iraqi Kurdistan Ministry of Housing and Transportation estimates traffic on these same roads to be 5,000 to 8,000 vehicles per day [10]. Motorization can indicate increased exposure, and if not accompanied by road safety legislation and adequate enforcement, may also indicate increased risk. Increased motorization was associated with increased rates of traffic fatalities in Germany following reunification in 1989 to 1991 and during times of economic progress in Nepal between 1981 and 2003 and Cambodia between 2006 and 2010 [8, 11, 12]. Estimates from the region with both annual numbers of road traffic fatalities and vehicles registered are unavailable.

While changes in motorization likely play a significant role in road traffic trends, whether elevated rates of road traffic in Iraq can also be attributed to the ongoing conflict has not been investigated. In Iraq, suicide car bombs and roadside improvised explosive devices (IEDs) are common mechanisms of warfare [13, 14]. Motor vehicles, including trucks, minibuses, fuel tankers, bikes, and cars, have been used as vehicles for suicide attacks. The effect of these explosives on road traffic, however, is not obvious. Given the frequency of incidents on roadways, we may anticipate civilians to avoid non-essential use of the roads, and therefore minimize time at risk of road traffic injury. However, destroyed road infrastructure and poor maintenance particularly in areas of greatest insecurity may result in increased risk to road users. Investigators have attempted to document the direct fatalities resulting from these explosives, but the indirect effects from destroyed road infrastructure and changes in driving behaviors are not well documented [15–17].

Iraq and global partners are investing in improving Iraq’s road infrastructure and safety. In 2013, the World Bank, in collaboration with the government of Iraq and the Islamic Development Bank approved the Transport Corridors Project, which has an estimated total cost of US$1.2 billion for road rehabilitation and new construction [18]. The World Bank estimates the project will reduce traffic fatalities by nearly 25 % on two national transport corridors [19]. Iraq has also made a commitment to road safety and publicly launched the Decade of Action for Road Safety 2011–2020 aimed at reducing road traffic fatalities [20].

Understanding the implications of modern warfare on other injuries, such as road traffic has important implications for understanding the burden of conflict on public health. Given the context of global investment in road infrastructure in Iraq, there is an even greater need for reliable and updated data on RTI fatalities. The few published studies examining road traffic injuries in Iraq have a limited geographic scope [21, 22]. In a recent correspondence in *The Lancet*, Al Saad and Sondorp described the paucity of reliable data on the incidence of road traffic injuries in Iraq and called for “more accurate cause-specific data” to address the growing public health threat posed by traffic accidents [8]. Other researchers from the region have also noted a scarcity of injury mortality data from Arab countries [23].

This study reviews data from the recently established Iraq Injury Mortality Surveillance System to examine the epidemiological pattern of road traffic fatalities in Iraq during a period characterized by a resurgence of violence. The system provides cause-specific data on road traffic fatalities to be used by partners to best target transportation programs in a conflict-affected country.

## Methods

**Fig. 1:**
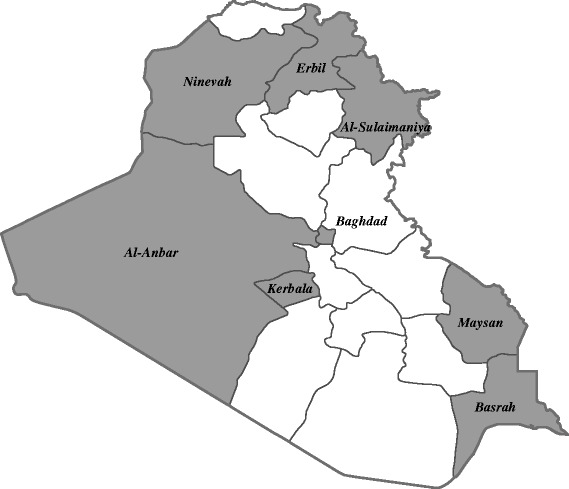
Map of selected governorates contributing injury surveillance data in Iraq

Data on fatal injuries caused by road traffic injuries between January 1, 2010 and December 31, 2013 were obtained from the Injury Mortality Surveillance System operated by the Iraqi Ministry of Health (MoH). Beginning January 1, 2010, this surveillance system began collecting data on deaths from injury reported by coroner offices in eight of the eighteen governorates of Iraq: Baghdad, Al-Anbar, Basrah, Erbil, Kerbala, Maysan, Ninevah, and Al-Sulaimaniya (Fig. 1). These governorates were selected by the Iraqi MoH as pilot governorates for Injury Surveillance based on convenience and willingness of coroner offices to participate in surveillance data collection. The selected governorates include two governorates in Kurdistan region (Erbil, Al-Sulaimaniya) in the northeast, as well as governorates in the northwest (Ninevah), central (Baghdad, Al-Anbar, Kerbala), and southern (Basrah, Maysan) regions of the country. Selected governorates include both areas with higher rates of insurgency related fatalities, such as Baghdad, Al-Anbar, and Ninevah, as well as areas that have experienced lower rates of insurgency related fatalities [24]. The Iraqi MoH and the Kurdistan Regional Government Ministry of Health initiated the system with technical support from the World Health Organization and the Centers for Disease Control and Prevention.

Trained clerks at the coroner offices collected data on fatal injuries using a standardized surveillance form. Information collected included the date and location of incident, victim demographics, death certificate number and date of issue, mechanism of injury, and circumstances of the incident. This information was ascertained from identification found with the body, post mortem examinations and police reports, and through interviews with family members. Completed surveillance forms were transmitted to the MoH for aggregate analysis. In Iraq, Law No 148 makes compulsory the registration of all births and deaths [25, 26]. Regulations require fatalities resulting from injury be reported to the coroner offices for an investigation of the death prior to issuing a death certificate. Insurance claims and other succession rights all require a death certificate [25]. Previously published studies in Iraq have demonstrated that death certificates were issued throughout the conflict; 81–92 % of reported deaths could be verified with death certificates [15, 17, 27].

The case definition used by the Iraqi Injury Surveillance System included all persons who died within thirty days of an external injury, including both intentional and unintentional injuries. Out of all injury fatalities documented at the coroner office, we analyzed those for which the primary mechanism of injury was recorded as road traffic. Road traffic fatalities were classified according to the type of road user.

We present rates per population as well as per vehicle. Population estimates used are annual projections published by the Iraq Central Organization for Statistics and Information Technology (COSIT) calculated based on the most recent available census (1987 for the Kurdish governorates and 1997 for the other six governorates) [28]. Number of vehicles includes all private sector motor cars registered at General Directorate of Traffic in 2011 as reported by COSIT [9]. The number of kilometres travelled for each mode of transportation, a commonly used metric of exposure, was not available. Rates per number of vehicles are considered a reasonable proxy to the number of vehicle kilometres travelled [29]. Fatalities per population provide an aggregate measure of risk.

The database was checked for duplicate entries by comparing victim demographics, the time and location of incident, and mechanism of injury. Descriptive analysis was performed using STATA statistical software (version 11.2). The Institutional Review Board of the Centers for Disease Control and Prevention determined this study to be “non-research” because it entailed secondary analysis of routinely collected public health surveillance data. Personal identifiers were not included in the final dataset used for analysis.

## Results

A total of 7,976 road traffic fatalities were documented between January 2010 and December 2013 in the eight Iraqi governorates under surveillance, 9.9 per 100,000 population per year. Table 1 presents annual trends in terms of the total number and population-based rates of road traffic fatalities as well as the proportion of road traffic fatalities among fatal injuries of any cause recorded in coroner offices, by year and governorate.

**Table 1:** Number and incidence of RTI fatalities, and proportion of RTI among all cause injury deaths, by year and governorate, Iraq 2010–2013

	2010			2011			2012			2013			Total		
	No	Rate^a^	%^b^	No	Rate^a^	%^b^	No	Rate^a^	%^b^	No	Rate^a^	%^b^	No	Rate^a^	%^b^
Governorate															
Erbil	257	16.3	31.6	341	21.1	41.5	343	20.7	44.0	308	18.1	39.3	1249	19.1	39.1
Maysan	114	12.0	33.0	168	17.3	34.1	157	15.7	30.7	171	16.7	30.7	610	15.5	32.0
Kerbala	125	12.0	40.3	177	16.6	48.4	173	15.8	45.5	182	16.2	46.1	657	15.2	45.3
Al-Sulaimaniya	239	13.0	32.2	282	15.0	39.3	262	13.6	40.5	277	14.0	41.1	1060	13.9	38.1
Al-Anbar	157	10.3	23.4	186	11.9	22.8	193	12.1	24.9	241	14.7	21.1	777	12.3	22.8
Baghdad	751	10.9	26.5	617	8.7	25.8	710	9.8	25.8	396	5.3	11.6	2474	8.6	21.7
Ninevah	198	6.2	15.3	231	7.1	19.3	264	7.9	20.3	219	6.4	10.0	912	6.9	15.2
Basrah	44	1.8	7.5	91	3.6	13.5	84	3.2	12.4	18	0.7	3.0	237	2.3	9.3
Total	1885	9.7	24.8	2093	10.5	28.0	2186	10.7	27.9	1812	8.6	18.6	7976	9.9	24.4

^a^Rate per 100,000; annual population projections from the most recent census published by the Iraqi Central Statistics Organization (COSIT)

^b^% refers to fatal injuries from RTIs as a percentage of all fatal injuries reported at the coroner office

**Fig. 2:**
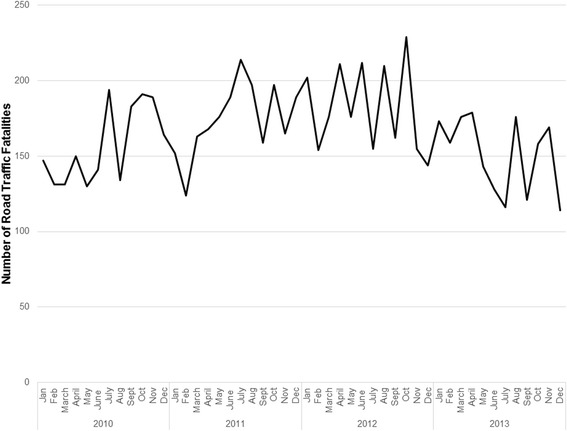
Number of road traffic fatalities in selected governorates of Iraq by month, 2010–2013

The number and rate of road traffic fatalities increased slightly between 2010 and 2012 with minor year on year fluctuations by governorate (Table 1). We observed an overall decrease in the number and rate of RTI fatalities in 2013, primarily due to a decline in Baghdad governorate. Baghdad governorate had the highest number of road traffic fatalities; however, the rate of fatalities was below average and decreased by 46 % from 2012 to 2013. The largest increase in incidence occurred in Al-Anbar from 10.3 per 100,000 in 2010 to 14.7 per 100,000 in 2013, a 43 % change. Al-Anbar was the only governorate with consistent year on year growth. The overall proportion of RTI fatalities among all cause injury fatalities followed a similar trend of a slight increase from 2010 to 2012 followed by a decline in 2013. No pronounced seasonality patterns were observed (Fig. 2).

The differences in road traffic fatality rates by governorate generally remained consistent through the period under surveillance. On average, Erbil experienced the highest rate of RTI fatalities for 2010–2013 (19.1 per 100,000 population per year). Road traffic fatalities caused the highest proportion of all cause injury fatalities in Kerbala in all four years (45.3 % overall). In Basrah, both the rates of road traffic fatalities (2.3 per 100,000 population per year) and proportion of RTI fatalities among all cause injury fatalities (9.3 %) were lowest compared with the other governorates.

Population based rates do not account for the notable differences in vehicle ownership by governorate. To account for differences in exposure, we present fatality rates per registered vehicle as of 2011, the latest year with available car registration data. The rate of RTI fatalities per 10,000 vehicles in 2011 was highest in Maysan (47.4), followed by Kerbala (27.2), Al-Sulaimaniya (14.3), Al-Anbar (10.1), Erbil (8.6), Ninevah (7.8), Basrah (5.1) and Baghdad (5.0). Overall, there were an estimated 8.1 fatalities per 10,000 vehicles in these governorates.

**Fig. 3:**
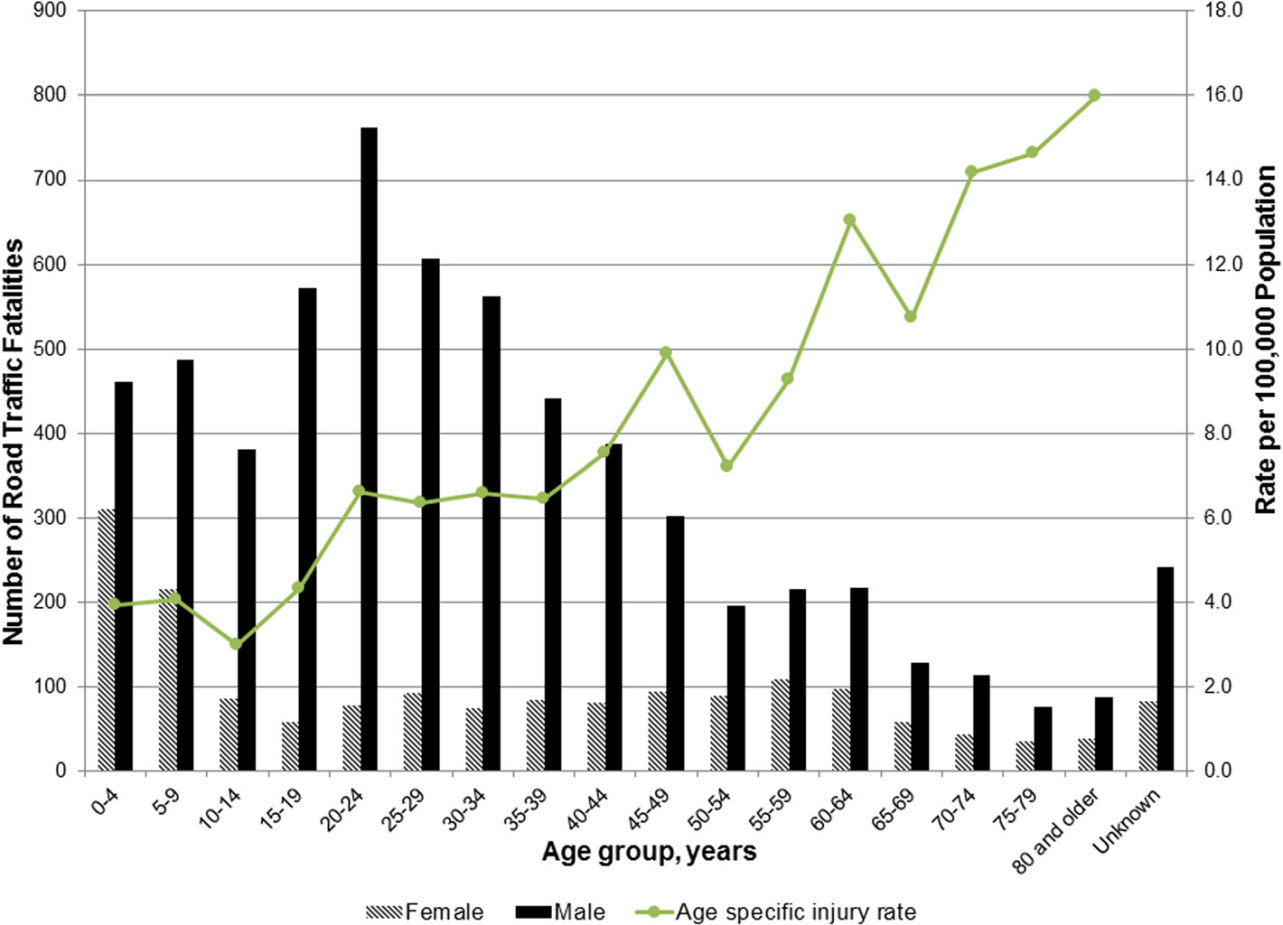
Age and sex distribution of road traffic fatalities in selected governorates of Iraq, 2010–2013^*±^. * Rates include males, females and fatalities with unknown gender. Number of fatalities with unknown gender [6 (0.08 %)] not shown. ^±^ Population data used for rates are 2011 projections from the Iraq Central Organization for Statistics and Information Technology (2011) [18]

The number of road traffic fatalities was greatest among adult males. Overall, 6,238 (78.2 %) fatalities were among males and 1,732 (21.7 %) fatalities were among females. 5,378 (67.4 %) road traffic fatalities were adults and 2,272 (28.5 %) were children under 18 years of age. The age of 326 (4.1 %) fatalities was unknown. The mean age of fatalities with known age was 30.5 years. When analyzed by 5-year age groups (Fig. 3), the highest numbers of RTI fatalities in males were in age groups between 15 and 34 years of age; males of this age range accounted for nearly a third (31.4 %) of all road traffic fatalities. In females, the 0–4 year age group had the greatest number of fatalities. The number of RTI fatalities was higher for males than females for all 5-year age groups (Fig. 3). The ratio of males to females was the highest in the 20–24 year age group (9.8:1); even among children under five there were substantially more males than females (1.5:1).

**Fig. 4:**
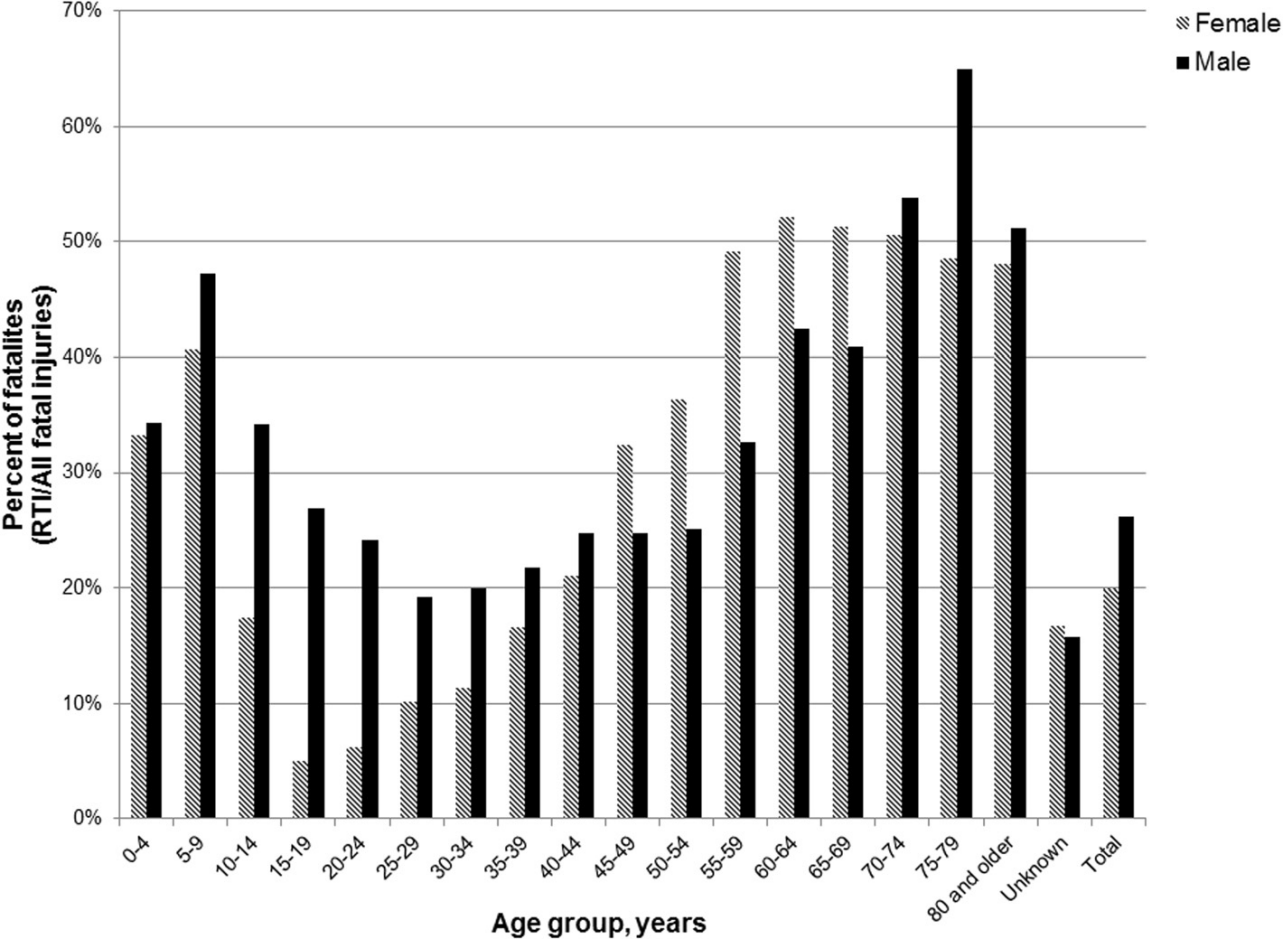
Percent of road traffic fatalities among all fatal injuries, by age and sex, 2010–2013. *Not shown: 142 (0.4 %) fatalites with unknown gender

Overall, RTIs were the cause of 24.4 % of all fatal injuries. Road traffic fatalities represented more than half of all fatal injuries among those aged 70 years and older (Fig. 4). Age specific fatality rates were also highest among these older age groups (Fig. 3). The proportion of RTI fatalities among all cause injury fatalities was higher among males (26.2 %) than among females (20.0 %), except for five year age groups between 45 and 69 years of age (Fig. 4).

The majority of road traffic fatalities were either pedestrians (49.2 %) or motorists (passenger or driver) (46.3 %). Fatalities among other types of road users were less common: motorcyclists, bicyclists, and other types of road users accounted for 3.7 %, 0.4 % and 0.4 % of all RTI fatalities, respectively. Table 2 presents the distribution of fatalities by the type of road user. Among females and children, the majority of road traffic fatalities were pedestrians (56.6 % and 69.0 %, respectively). The distribution of road user types among RTI fatalities differed by governorate. In Al-Anbar (72.2 %), Erbil (73.6 %), Maysan (74.9 %), Ninevah (57.9 %), and Al-Sulaimaniya (52.2 %) the majority of fatalities were among motorists. On the other hand, in Baghdad 84.5 % of RTI fatalities were pedestrians. In Kerbala, motorcyclists comprised 9.3 % of fatalities, nearly three times the average for the eight governorates. With few exceptions, the distribution of injuries by mechanism did not change substantially from year to year.

**Table 2:** Distribution of RTI fatalities by type of road user, Iraq 2010–2013

	Pedestrian	Car	Motorcycle	Other/Unknown	Total
	Number (Row %)	Number (Row %)	Number (Row %)	Number (Row %)	number
Total fatalities	3,925 (49.2)	3,695 (46.3)	292 (3.7)	64 (0.8)	7,976
Gender					
Female	981 (56.6)	730 (42.1)	15 (0.9)	6 (0.3)	1,732
Male	2,940 (47.1)	2,963 (47.5)	277 (4.4)	58 (0.9)	6,238
Unknown	4 (66.7)	2 (33.3)	0	0	6
Age					
Child (under 18 years)	1,567 (69.0)	621 (27.3)	54 (2.4)	30 (1.3)	2,272
Adult	2,089 (38.8)	3,019 (56.1)	236 (4.4)	34 (0.6)	5,378
Unknown	269 (82.5)	55 (16.9)	2 (0.6)	0	326
Governorate					
Basrah	110 (46.4)	116 (48.9)	9 (3.8)	2 (0.8)	237
Al-Anbar	191 (24.6)	561 (72.2)	19 (2.4)	6 (0.8)	777
Baghdad	2090 (84.5)	333 (13.5)	46 (1.9)	5 (0.2)	2,474
Erbil	257 (20.6)	919 (73.6)	62 (5.0)	11 (0.9)	1,249
Kerbala	354 (53.9)	228 (34.7)	61 (9.3)	14 (2.1)	657
Maysan	130 (21.3)	457 (74.9)	15 (2.5)	8 (1.3)	610
Ninevah	363 (39.8)	528 (57.9)	11 (1.2)	10 (1.1)	912
Sulaimaniya	430 (40.6)	553 (52.2)	69 (6.5)	8 (0.8)	1,060
Year					
2010	894 (47.4)	865 (45.9)	105 (5.6)	21 (1.1)	1,885
2011	988 (47.2)	1036 (49.5)	59 (2.8)	10 (0.5)	2,093
2012	1213 (55.5)	891 (40.8)	68 (3.1)	14 (0.6)	2,186
2013	830 (45.8)	903 (49.8)	60 (3.3)	19 (1.0)	1,812

## Discussion

Our study provides the first analysis of cause-specific demographically disaggregated data on fatal road traffic injuries in a conflict-affected country. Between 2010 and 2013, we documented nearly 8,000 road traffic fatalities, an annual rate of between 8.6 and 10.7 deaths per 100,000 population. The rates calculated using the numbers of road traffic fatalities from all 18 governorates reported by COSIT (7.7 per 100,000 population in 2010; 8.1 in 2011; 9.2 in 2012; and 8.4 in 2013) are similar, albeit slightly lower, than our data from the eight governorates and demonstrate the same trend [30–32]. These surveillance data may therefore be used to complement COSIT, providing real time trends to be used for public health action in advance of the nationally published estimates.

The rates of road traffic fatalities in Iraq during 2010-13 have been relatively stable, with slight increase from 2010 to 2012 followed by a decline in 2013. The 2013 decline was due mostly to decrease in the number of injuries seen in Basrah and Baghdad. The number and rate of road traffic fatalities was nearly halved in Baghdad between 2012 and 2013, driving down the overall rate of road traffic fatalities in the governorates under surveillance. The trends in proportion of RTI fatalities among all cause injury fatalities showed the same pattern. These trends appear to be negatively correlated with trends in conflict-related fatalities. Between 2010 and 2012, as road traffic fatalities increased, Iraq experienced a relative reduction in violence. The number and rates of fatalities attributed to conflict and conflict-related explosions doubled in 2013, as RTI rates declined [24].

In Iraq, recent conflict is isolated to only a few governorates; other governorates have remained relatively unaffected by insurgency activity [24, 33]. This allows us to isolate the impact of violence by comparing trends in road traffic in high conflict governorates relative to overall trends. According to previous analysis of explosion-related fatalities in these select governorates, 95 % of all conflict-related explosion fatalities recorded in eight governorates under surveillance were concentrated in three governorates—Baghdad, Ninevah, and Al-Anbar [24]. Explosion-related fatalities increase substantially in these three governorates from 2012 to 2013. RTI fatalities, on the other hand, decreased in Baghdad and Ninevah but increased in Al-Anbar during the same period. These trends may suggest that conflict has differentially affected road traffic in these high conflict areas, potentially resulting from differences in levels of road infrastructure damage, driving or road utilization. The relationship between insurgency and road traffic fatalities may also relate to the volume of traffic and number of roadways, such that infrastructure damage has a greater impact in a desert governorates such as Al-Anbar than a more urban governorate such as Baghdad. Data on the circumstances of the injury, such as proximity of the incident to an area of recent conflict or the road condition at the site of the incident, would help better understand the impact of conflict.

Annual trends in rates per registered vehicles could not be calculated in Iraq as vehicle registration data disaggregated by governorate is not published annually in Iraq. However, available data demonstrate that differences in number of fatalities by governorate coincide with marked differences in vehicle ownership by governorate. The highest fatality rate per population was in Erbil, the governorate with the most registered cars per capita [9]. The rate per registered vehicle, a measure of exposure, is therefore relatively low in Erbil, a fifth the fatality rate per vehicle recorded in Maysan. Particularly in countries like Iraq, where a substantial portion of the population are not road users, rates that account for differences in vehicle ownership, kilometers travelled, or other measures of exposure, help understand the true burden of road traffic fatalities. Kilometers driven and hours of exposure are likely more affected by conflict than vehicle ownership, however are not available in Iraq or most conflict-affected countries.

Approximately eight out of ten road traffic fatalities in Iraq were males. The proportion of males among RTI fatalities reported from Iran (79 %), Jordan (81 %), Turkey (77 %), Lebanon (77 %), United Arab Emirates (89 %), and Egypt (80 %) show very similar patterns, suggesting that males are disproportionately affected [1, 34]. Globally, more than three-quarters of road traffic fatalities are male [1]. While sex distribution of RTI fatalities in Iraq is similar to global and regional patterns, the age distribution is somewhat distinct. Among males, the number of road traffic fatalities peaked among the age groups between 15 and 34 years of age; however among females, the number of fatalities was highest in the under-five age group. A high proportion of injuries among young adults has been documented in many contexts; however, the high number of fatalities among young children is a notable peculiarity in demographic profile of RTI injuries in Iraq [1, 34, 35]. WHO estimates that globally fewer than 5 % of road traffic deaths occur among children under five years, a contrast to Iraq where this age group represents approximately 10 % of all road traffic fatalities and 18 % of fatalities among females [1]. Children under five years represent only approximately 3 % of RTI fatalities in Iran [34]. This difference may be explained in part by the age structure of the Iraqi population where children under five years represent approximately 15 % of the population, a greater proportion than in the global population (8 %) or any other country in the region [36, 37]. Research from the West Bank and Gaza Strip has suggested that increased risk among this age group may be attributed to children playing in streets where designated safe play spaces are not available [23]. The absence of child restraint laws may also contribute to the increased risk [1, 7]. Another interesting observation from our data is a high rate and proportion of RTI among all cause injury fatalities among older adults. For people 70 years of age and older, RTI fatalities caused more than half of all injury fatalities. Age specific fatality rates generally increased with age.

We found that nearly half of all road traffic fatalities were among pedestrians, a proportion notably higher than in neighboring countries. According to the Turkish Statistical Institute, 19 % of RTI fatalities in Turkey were pedestrians in 2008 [38]. Surveillance data from Egypt suggests approximately 24 % of road traffic fatalities were pedestrians in 2009 [34]. Similarly, national death registration data from Iran indicated that 29 % of RTI fatalities were among pedestrians in 2005 [35]. Additionally, our data show that women and children in Iraq were more likely to be involved in fatal pedestrian accidents than any other type of road traffic accident. Nearly 70 % of children under 18 years of age and 57 % of women who were fatally injured in a road traffic accident were pedestrians. Men killed by RTIs were equally as likely to be a pedestrian as a car occupant. This could reflect differences in modes of travel among women and children compared with men. For example, car ownership is lower among women in other regional countries, including Iran [38]. The high proportion of pedestrian fatalities in Iraq warrants further studies of the circumstances of the incidents leading to pedestrian deaths.

Fatalities among motorcyclists accounted for fewer than 4 % of all road traffic deaths. Motorcycle injuries represent a greater proportion of road traffic fatalities in other countries in the region, including Iran where motorized 2-wheel vehicles accounted for 23 % of reported road traffic fatalities in 2010–2011 [1] and Egypt where motorcyclists represented 13 % of reported road traffic fatalities in 2009 [34]. Number of motorcyclist fatalities remained consistently low in Iraq over the surveillance period. This finding is surprising given the legal environment; Iraq has no mandated helmet standard and national motorcycle legislation is not yet well enforced [1, 7]. The lower proportion of motorcycle related deaths in Iraq is likely related to lower use of motorcycles, although data on motorcycle registration in Iraq are limited [1].

While data presented here are from governorates selected based on convenience, we suggest these data provide useful insight for Iraq more generally. The eight governorates that participated in pilot surveillance project represent northern, central, and southern parts of Iraq. On average, 72.8 % of the population per governorate lives in urban centers in the eight governorates included in surveillance (range: 48.4–87.2 %), similar to the national average (65.9 %; range 43.7–87.2 %) [39]. These governorates are however, more developed and have more vehicles on average. According to the World Health Organization Joint Analysis Unit (JAU), on average 10.9 % of the population are living on less than $2.5 USD per day in the select governorates (range 0.3–26.0) compared to 13.1 % in Iraq nationally (range: 0.3–37.8) [39]. The number of registered private motor cars per person in selected governorates was 130.1 per 1,000 people (range 36.5–244.7) compared to Iraq nationally (105.1; range 34.9–244.7) [9, 28].

This study is subject to several limitations. First, some RTI fatalities may not be reported to the surveillance system. While death registration is mandatory in Iraq, there remains the possibility of unreported deaths. This risk is likely greatest in areas of conflict and insecurity, where ongoing data collection puts coroner staffs in personal danger. To minimize risk of traveling between sites, data entry is performed in each governorate and digitally transferred to the national ministry for aggregate analysis. With this and other precautions, coroners were able to consistently report data from all sites during the period reviewed in this study. However, as insecurity increased in 2014, coroners stopped reporting surveillance data consistently in areas with greatest insurgency activity: Al-Anbar and Ninevah. Additionally, the system collects only a limited number of RTI-related variables since it was designed to be simple with limited burden on reporting facilities. Variables such as road conditions at incident sites, and time/place proximity to recent insurgency activity, as well as more conventional risk factors such as seat-belt use, helmet use, and controlled substance use are needed to better understand the road traffic data presented here, including the link between road traffic fatalities and conflict. The addition of such modifiable behavior variables would also be useful in informing intervention policies. More focused studies may also be needed to build a nuanced understanding of risk factors, better elicit the circumstances surrounding the cause of the accident, and account for differences in exposure and risk.

## Conclusions

In conclusion, the data from the Iraqi Injury Mortality Surveillance System provided a detailed picture of the burden of road traffic fatalities in Iraq by governorate, demographic group and mechanism. The highest numbers of road traffic fatalities were among young men. Pedestrians and other vulnerable road users accounted for half of road traffic fatalities and for even a greater proportion among women and children. Road traffic fatalities in the eight governorates under surveillance declined from 2012 to 2013, coinciding with the resurgence of conflict in Iraq.

Consistent with previous research, our data suggests that despite the conflict, road traffic fatalities continue to contribute substantial to the injury profile of Iraq [40]. The Iraqi Ministry has pledged to a reduction in road traffic fatalities by 2020 in accordance with the Decade of Action for Road Safety 2011–2020 [19]. The Iraq Injury Mortality Surveillance System can serve a vital role in tracking progress towards this goal. The benefits of injury surveillance are numerous — enabling the quantification of injuries, describing demographics of those affected, and tracking trends over time.

Data collection is ongoing and, as of mid-2013, expanded in coverage to all 18 governorates. Coroner offices involved in this eight governorate pilot supported the national scale-up to ensure standardization of data collection nationally.

Accurate, up to date information is essential for advising road safety policies, particularly given the fluidity of the changing conflict. As surveillance data on fatalities from insurgency activity and road traffic are currently reported by governorates on a monthly basis, more frequent analysis is possible and would increase the utility of the data. National plans now encourage regular meetings of coroners from different governorates to review the data, a lesson from the eight governorate pilot. Surveillance data can serve as a useful tool in these efforts to provide information for evidence-based prevention strategies to reduce the burden of road traffic injuries.

### Ethics approval

This study was exempted from review by the Institutional Review Board of the Centers for Disease Control and Prevention as the primary intent was surveillance and was determined to not involve human subject research. The study constitutes a secondary analysis of surveillance data routinely collected for programmatic purposes.

## Abbreviations

COSIT: Central Organization for Statistics and Information Technology
EMR: Eastern Mediterranean Region
GDP: Gross Domestic Product
JAU: Joint Analysis Unit
MOH: Ministry of Health
RTI: Road traffic injuries
WHO: World Health Organization

## References

[1] World Health Organization (2013). Global status report on road safety 2013: supporting a decade of action.

[2] Lozano R, Naghavi M, Foreman K, Lim S, Shibuya K, Aboyans V (2012). Global and regional mortality from 235 causes of death for 20 age groups in 1990 and 2010: a systematic analysis for the Global Burden of Disease Study 2010. Lancet.

[3] Global Burden of Disease Study 2013 (2014). Global Burden of Disease Study 2013 (GBD 2013) Age-Sex Specific All-Cause and Cause-Specific Mortality 1990–2013.

[4] Bachani AM, Zhang XJ, Allen KA, Hyder AA (2014). Injuries and violence in the Eastern Mediterranean Region: a review of the health, economic and social burden. East Mediterr Health J.

[5] World Health Organization (2013). Road safety in the Eastern Mediterranean Region: Facts from the Global Status Report on Road Safety.

[6] Soori H, Hussain SJ, Razzak JA (2011). Road safety in the Eastern Mediterranean Region--findings from the Global Road Safety Status Report. East Mediterr Health J.

[7] World Health Organization (2009). Global status report on road safety : time for action.

[8] Al Saad NA, Sondorp E (2013). Road traffic injuries in Iraq. Lancet.

[9] Central Organization for Statistics and Information Technology (COSIT). Statistics of Private Sector Motorcars That Registered at General Directorate of Traffic until 31/12/2011. http://cosit.gov.iq/en/trans-comm-statistics. Accessed 7 Feb 2016.

[10] A Strategic Plan Key to Development: Q&A with Kamaran Ahmed Abdullah, Minister of Housing and Reconstruction. The Review Kurdistan. Kurdistan Region of Iraq: Invest in Group. 2013. http://www.investingroup.org/files/the_review-kurdistan_region_of_iraq-march_2013.pdf. Accessed 1 Aug 2015.

[11] Parker EM, Ear C, Roehler DR, Sann S, Sem P, Ballesteros MF (2014). Surveillance of road crash injuries in Cambodia: an evaluation of the Cambodia Road Crash and Victim Information System (RCVIS). Traffic Inj Prev.

[12] Poudel-Tandukar K, Nakahara S, Poudel KC, Ichikawa M, Wakai S (2004). Traffic fatalities in Nepal. JAMA.

[13] Brevard S, Champion H, Katz D. Weapons Effect. In: Savitzky E, Eastridge B, editors. Combat Casualty Care: Lessons Learned from OEF and OIF: Office of the Surgeon General Department of the Army, United States of America. 2012. p. 43–83.

[14] Dodd H, Perkins R. An Explosive Situation: Monitoring explosive violence in 2012: Action on Armed Violence. 2013.

[15] Hagopian A, Flaxman AD, Takaro TK, Esa Al Shatari SA, Rajaratnam J, Becker S (2013). Mortality in iraq associated with the 2003–2011 war and occupation: findings from a national cluster sample survey by the university collaborative iraq mortality study. PLoS Med.

[16] Iraq Body Count (IBC). Iraqi deaths from violence 2003–2011: Analysis and overview from Iraq Body Count. 2012. http://www.iraqbodycount.org/analysis/numbers/2011/. Accessed 1 Sept 2015.

[17] Burnham G, Lafta R, Doocy S, Roberts L (2006). Mortality after the 2003 invasion of Iraq: a cross-sectional cluster sample survey. Lancet.

[18] The World Bank (2013). Iraq - Transport Corridors Project.

[19] The World Bank. Better and Safer Roads in Iraq will Boost Regional Trade, Save Lives and Promote Citizens Engagement. Washington DC. 2013. http://www.worldbank.org/en/news/press-release/2013/12/19/better-safer-roads-regional-trade-citizens-engagement. Accessed 1 Aug 2015.

[20] World Health Organization. Iraq committed to improving road safety. 2011. http://www.emro.who.int/violence-injuries-disabilities/countries/iraq-roadsafety.html. Accessed 1 Aug 2015.

[21] Murad MK, Issa DB, Mustafa FM, Hassan HO, Husum H (2012). Prehospital trauma system reduces mortality in severe trauma: a controlled study of road traffic casualties in Iraq. Prehosp Disaster Med.

[22] Nakshabandi MM (2007). Casualties and Deaths From Road Traffic Accidents in Dohuk, Iraq. Dohuk Med J.

[23] Shaheen A, Edwards P (2008). Flying bullets and speeding cars: analysis of child injury deaths in the Palestinian Territory. East Mediterr Health J.

[24] Bilukha O, Leidman E, Sultan AS, Hussain S (2015). Deaths due to intentionalexplosions in selected governorates of Iraq from 2010 to 2013: prospective surveillance. Prehosp Disaster Med.

[25] Al-Rabie AS. Registration of Vital Events in Iraq. Technical Papers, 1980:1–5. http://www.cdc.gov/nchs/data/isp/010_Registration_of_%20Vital_Events_in_Iraq.pdf. Accessed 1 Aug 2015.

[26] Iraqi Legal Library. Medico-Legal law no. (37) for the year 2013. 2013. http://www.iraq-lg-law.org/ar/taxonomy/term/108. Accessed 1 Aug 2015.

[27] Roberts L, Lafta R, Garfield R, Khudhairi J, Burnham G (2004). Mortality before and after the 2003 invasion of Iraq: cluster sample survey. Lancet.

[28] Central Organization for Statistics and Information Technology (COSIT). Section 2: Population Census. 2012. http://cosit.gov.iq/AAS/section_2.php. Accessed 1 Aug 2015.

[29] Hakkert AS, Brainmaisert L. The uses of exposure and risk in road safety studies. SWOV Institute for Road Safety Research. 2002. http://www.swov.nl/rapport/R-2002-12.pdf. Accessed 29 Dec 2015.

[30] Central Organization for Statistics and Information Technology (COSIT). Number of Casualties by Road Traffic Accidents by Sex for the Years 2004-2011. 2011. http://www.cosit.gov.iq/AAS13/TRANSPORTATIONANDCOMMUNICATIONSTATISTICS6/trans11.htm. Accessed 1 Aug 2015.

[31] Central Organization for Statistics and Information Technology (COSIT). Press Release: Statistic of Traffic Accident Recorded for 2013. Release No 26 ed. 2014. http://www.cosit.gov.iq/images/press_release/en/pdf/press26_2014.pdf. Accessed 1 Aug 2015.

[32] Central Organization for Statistics and Information Technology (COSIT). Press Release: The Recorded Traffic Accidents Increase in 2012. Release No 16 ed. 2012. http://www.cosit.gov.iq/images/press_release/en/pdf/press16.pdf. Accessed 1 Aug 2015.

[33] Iraq Body Count (IBC). Iraq 2014: Civilian deaths almost doubling year on year: An overview of the year’s violence. 2015. https://www.iraqbodycount.org/analysis/numbers/2014/. Accessed 1 Sept 2015.

[34] World Health Organization (WHO). Injury Surveillance: A tool for decision-making; Annual injury surveillance report Egypt, 2009. WHO-EM/HLP/067/E. Regional Office for the Eastern Mediterranean, Arab Republic of Egypt: Ministry of Health. 2009. http://applications.emro.who.int/dsaf/dsa1087.pdf. Accessed 1 Aug 2015.

[35] Bhalla K, Naghavi M, Shahraz S, Bartels D, Murray CJ (2009). Building national estimates of the burden of road traffic injuries in developing countries from all available data sources: Iran. Inj Prev.

[36] Central Organization for Statistics and Information Technology (COSIT). Popylation of Iraqs Projections by Age Groups, Social Origin & Gender 2011 (sic). 2011. http://www.cosit.gov.iq/en/industrial-statistics/small-facilities?id=559. Accessed 1 Aug 2015.

[37] United Nations Department of Economic and Social Affairs. World Population Prospects: The 2012 Revision. http://esa.un.org/wpp/excel-data/population.htm. Accessed 1 Aug 2015.

[38] Puvanachandra P, Hoe C, Ozkan T, Lajunen T (2012). Burden of road traffic injuries in Turkey. Traffic Inj Prev.

[39] United Nations Joint Analysis Unit. Iraq Information Portal. Governorate Profile. http://www.iau-iraq.org/gp/. Accessed 20 May 2015.

[40] Sosa LM, Bhatti JA (2013). Road traffic injuries in conflict areas. Inj Prev.

